# Transitions that Matter? Czechoslovakia’s Break up and Human Stature

**DOI:** 10.3390/ijerph16245050

**Published:** 2019-12-11

**Authors:** Joan Costa-Font, Lucia Kossarova

**Affiliations:** 1 Department of Health Policy, London School of Economics and Political Science, London WC2A 2AE, UK; 2 Department of Health Policy, London School of Economics and Political, London WC2A 2AE, UK; kossarova@gmail.com

**Keywords:** height, democracy, transition, secession, Czechoslovakia, Slovakia, Czech Republic

## Abstract

Changes in a population’s average stature are virtuous pointers of wellbeing which are sensitive to improvements in psychosocial environments during childhood. A major structural change that could have altered an environment during childhood is the transition from communist to a liberal democracy, and, more specifically, the meltdown of the Soviet bloc provides for a quasi-natural experiment. This paper examines the trends in heights in the Czech Republic and Slovakia before and after the transition and the subsequent break-up of the Czechoslovakian federation. We find that one additional year of exposure to a liberal democracy while growing up is associated with an increasing population stature of 0.28 cm among Slovaks and 0.15 cm among Czechs. We only find changes in stature among men who are more sensitive to environmental stress, especially at the lower end of the current socio-economic status. Results are robust to alternative datasets and measures of democracy.

## 1. Introduction

A child’s exposure to conditions that are less than optimal might impact the capacity to attain his or her height potential [1]. This is the case because physical stature reflects how a human organism fares during childhood and adolescence in its socio-economic and epidemiological environment [2]. As much as 20 percent of the variation in human stature is attributed to ‘environmental’ factors, both adverse and beneficial [3]. For instance, exposure to civil or military conflict between birth and adolescence exhibit reduced adult stature [4]. A question that emerges is whether structural reforms, such a country’s transition to a liberal democracy, which one would expect to impact on beneficial psychosocial environments (e.g., increasing social participation, equal rights, freedom of speech, stability, impacting on lower stress etc.), reflects in changes in human stature.

The meltdown of the Soviet bloc provides a natural experiment to test for the “fit through democracy” hypothesis [5], which states that extending the “franchise for all adults” enables reforms that bring about wellbeing. Democratization in such a context might have structurally reshaped minority inclusiveness, improved perceptions of safety and health information, and produced a cultural change in attitudes [6]. In examining the effects of German reunification, evidence suggests that although West Germans are taller than East Germans, since unification there has been convergence in heights between East and West German males but, paradoxically not among females [7]. The latter finding has yet to be better understood. Furthermore, it seems important to examine whether the latter findings can be made extensive to all Eastern European countries in the area of Soviet influence.

A priori it is not clear that everyone benefited from the transition to a liberal democracy given the elitist nature of democracy in Eastern Europe, and whether it affected human heights, as it would have to have influenced key periods of human growth [2]. The advantage of using height measures over other measures of wellbeing is that is mostly free of measurement error related to other individual characteristics [8]. However, from other studies [7] one can expect significant heterogeneity on the effect of gender and socio-economic status [9,10]

This paper draws on evidence from Czechoslovakia, a country that was in the Soviet bloc, but was broken up in the following few years, after the transition to democracy. Thus, it gave rise to a rare case in history where two large liberalisation forces coincided, often referred to as a “double bang” [11]. A country’s break up offers an opportunity to reshape each country’s institutions, reduce conflict, and more generally improve institutional quality [12,13]. Our paper contributes to the following:

First, we attempt to add a wider literature on democracy and health [14] which examines the benefits of the adoption of a liberal democracy. Democracies reduce instability [15], which can benefit children by reducing their levels of exposure to environmental stress. However, empirical evidence on this question is still scarce, and it should account for the fact that poor nutrition was a problem in Eastern Europe prior to the transition due to seasonal unavailability of certain foods. Another problem is that in many Eastern European countries, there was a deterioration in living standards before any visible improvements took place [16,17,18]. Hence, it is not even trivial that one should expect an effect overall. This paper contributes to the literature by documenting evidence of the height changes in the Czech Republic and Slovakia before and after the transition of liberal democracy and breakup. Further, we control for differences in observable socio-economic status (income) which captures individual specific changes in economic status alongside some other covariates. It adds to a growing literature that has documented an effect of democracy and female franchise on heights [19].

Second, we examine gender and income heterogeneity. Males are found to be more affected by environmental stress, are more sensitive to changes in the environment than females and exhibit a greater response to nutritional supplementation [20]. Similarly, studies examining social inequality in Eastern Europe suggest heterogeneous patterns of income and gender disparities after 1989 [21]. That is, they show that income inequalities declined consistently with other related evidence that finds that environmental shocks affect primarily the heights of children in low socio-economic conditions [22] This is important given that democracy might not benefit all equally. Individuals who were at the ‘elite’ of the previous regime (before the transition), might not exhibit comparable changes in stature as the rest. Komlos and Kriwy [7] find evidence of German unification on male but not female stature. Consistently, evidence suggests that transition to a liberal democracy in Eastern Europe has exerted small effects on gender inequality [23] Hence, it is an empirical question whether transition did affect women’s heights as much as it did men.

Third, the break-up of Czechoslovakia allows us to identify two different trajectories of reform which depart from comparable institutional conditions. This evidence adds to the literature on the effects of self-determination on health [24]. One of the potential concerns is that democracy is not a categorical variable, and hence we should not only measure the effects of exposure to a democracy while growing up but account for its quality, which we do by adjusting our exposure measured by the Polity IV index of each of such years of exposure.

The next section contains the background on the specific case study. Section three reports the data and methods. Section four contains the results, section five the robustness checks, and a final discussion section concludes the paper.

## 2. Czechoslovakia’s Double Bang

After World War II, in 1948, Czechoslovakia fell under the Soviet influence. The latter implied a ban on civil and political liberties alongside media censorship and economic dirigisme with the implementation of production plans and quotas. The regime lasted forty years until 1989 with only a small spell of the Prague spring when reform was attempted. Although initially, the steps taken in the two federations of Czechoslovakia were similar, in 1992 a peaceful secession process was designed by the two main community leaders to create two separate countries in 1993. The events of 1989 and 1992 can be regarded as a “double bang”, a rare case in history where two large forces coincided [11]. It was first a transition from centrally planned to a market economy and then the secession of Slovakia that happened virtually simultaneously. Some even suggest that it was a “triple transition”: Democratization, marketization, and a national transformation [25]. However, whilst marketisation can be examined by examining the effect of income and standards of living, the democratization and its subsequent reforms might produce wider effects on wellbeing that are not necessarily measured in traditional welfare indicators, such as changes in gender, income, and other inequalities, and access to welfare services that might not produce immediate effects on economic outcomes.

Already during the communist period, the Czech Republic and Slovakia differed in their level of economic and social development. After secession, the form and speed of the democratization and liberalization reforms gradually began to differ. The Czech Republic initially implemented aggressive economic reforms in combination with socio-economic entitlements and democracy. In contrast, in Slovakia, the first years after the break-up were characterized by a continuation of an authoritarian rule which left the country economically and politically isolated [26]. Slovakia was severely disadvantaged throughout the 1990s, but by 1998 the rapid progress in the Czech Republic slowed down and the reverse happened in Slovakia. The period between 1989 and 2004 is defined as a ‘transformation shock’ [26]. Hence, it is an empirical question whether such reforms produced desirable welfare effects on human stature. The remainder of the paper will be devoted to measuring such effects.

## 3. Data and Methods

### 3.1. The Data

This study uses as primary data from the 2003 World Health Survey (WHS), which is the baseline household survey for the health status of populations and outcomes related to investments and functioning of health systems. This data draws on slight difference sampling, but ensures a sample of about 1000 observations per country. Given that WHS has a relatively smaller sample for the Czech Republic—mainly due to non-response—an additional representative survey, namely the 2005 Eurobarometer survey 64.3 [27] is employed for robustness purposes.

The WHS samples all the adult population over age 18 years old using a probability sampling design either with single or multi-stage random cluster sampling. Individual probability sampling weights were available to adjust for the probability of selection into the sample 28] According to the WHS individual country reports provided by the WHO, the number of interviewed households was 935 in the Czech Republic and 1811 in Slovakia. According to the official WHS country report of the Czech Republic, the sample is representative of the population and follows the same procedure as the Slovak sample (see Appendix A). We identified some potential selection bias driven primarily by the low response rate of Czechs. The survey includes information individual’s height (in cm) as well as information on other important variables that are controlled for including education, income, rural or urban location, employment, and others. Table 1 below summarizes the main variables used in the analysis, and Table A1 and Table A2 in the Appendix A provide the descriptive statistics. The distribution of the respondents differs in only a small number of characteristics which are important to control for, especially for gender and age groups. There were somewhat more women and fewer men in the sample than the overall population (55.2% compared to 52%, and 44.8% compared to 48%, respectively). However, the WHS sample compared to the overall population for regional representation, ethnicity, family status, education, economic activity and employment, and household composition. Finally, it is important to mention that although the height data is self-reported, and hence there is a potential self-reporting bias, if there is a bias it is likely to affect both those exposed and not exposed to a liberal democracy.

The key explanatory variables are represented by the number of years a person has lived under democracy (damage) and independence (DemIndyage) before they reach 20 years of age. The average period under democracy was 5 years and the average tie under independence was 3.3 years, as reported in Table A1 in the Appendix A. The democracy measure (refers to individual records after 1989, starting at 1990) refers to individual records of people aged 18 to 33 in the year 2003 (birth cohorts 1970–1985) who, of their first 20 years, lived between 1 and 14 years under democracy (6 to 19 years under communism). All the older individuals recorded lived all of their first 20 years under communism. Similarly, for independence (1993), individuals aged 18 to 30 in the year 2003 (birth cohorts 1973–1985) lived, of their first 20 years, between 1 and 11 years as part of an independent country (or 9to 19 years as part of Czechoslovakia). Given that measuring the effect of a democracy with a dummy variable is a too crude assumption, we then controlled for the “quality” of the democratic years by means of adding the most well-accepted index of democracy, the so-called Polity IV institutionalized democracy variable (dempolity) after 1993 for independent Slovakia and Czech Republic. The score was used to weigh the years exposed to a liberal democracy. These weighted years were then added up to obtain an adjusted democracy variable. For both Slovakia and the Czech Republic, the scores were positive (7 and above) for the entire period under study, so the weights used were between 0.7 and 1. These weighted years were then added up to obtain an adjusted democracy variable.

Given that the dataset contained no income or wealth data, we employed a data reduction technique from a series of questions about the ownership of particular household objects (e.g., number of cars, TVs, rooms, ownership of phone, video camera, computer and access to internet) to estimate a measure of permanent income [29,30] In addition to gender and age groups, we have information about whether the individual is employed, and the language spoken, which can be a proxy for ethnic differences in the two countries.

### 3.2. Empirical Strategy

Our empirical strategy draws on classical height regressions estimated by ordinary least squares (OLS) to identify the association between exposure to democracy $(D_{i}$)—once we control for a number of covariates $(X_{i}$)—and population height ($H_{i}$),

(1) $$H_{i}=\;\delta D_{i}+\gamma X_{i}+\epsilon_{i}$$

*X_i_* is a vector containing the set of controls described below, and a constant; δ,γ are the coefficients of the slope parameters and the intercept; ε is the error term that follows the conventional properties; and *I* refers to the individual respondent. Democracy (*D_i_*) refers to the number of years under a democratic regime in its more straightforward definition. Alongside this, we estimate the effects of the exposure to democracy after independence, and exposure to democracy once each year is weighted by the “quality” using data from Polity IV. Finally, we have performed some robustness checks that help to disentangle the extent to which the association is robust to samples and measures.

## 4. Results

### 4.1. Preliminary Evidence

**Figure 1:**
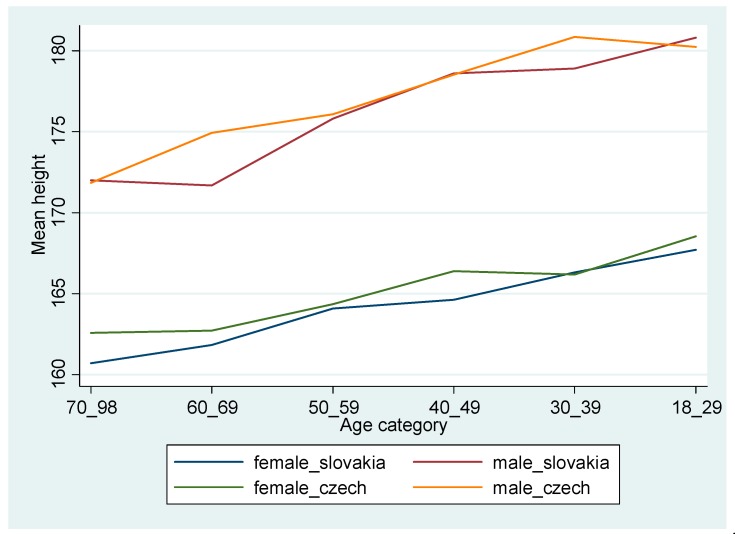
Height by age cohorts, gender and country in 2003. Source: [28].

Figure 1 displays an increasing trend in heights across the age groups. To make secular trends more visible, we report the younger groups at the latest point of the X-axis. As expected, older generations are shorter than the younger ones in both countries, and there is a gender gap in heights that persists across different age groups. Overall, the difference over age cohorts appears to be more important than the difference between the two countries.

Table 2 displays estimates of the average heights between men and women as well as between the Slovak and the Czech population by age groups. Consistent with Figure 1, we find an increasing trend across age cohorts. The range for Slovak males between the oldest and the youngest age groups is as much as 8.79 cm, followed by Czech men (8.41 cm), Slovak women (6.99 cm), and Czech females (5.97 cm). We find sizeable and significant height differences between the two countries for men aged 30–39 years (born 1964–1973) and women aged 40–49 years (born between 1954–1963).

Table 3 displays the mean height across income terciles by country and gender. We find that the heights distribution is heterogeneous both across income terciles within and between countries, but the variation is more significant for men. The average height of Slovak and Czech women varied less than 0.2 cm across income terciles; the difference was almost twice among men in both countries.

### 4.2. Baseline Results

Next, we report in Table the baseline estimates of Equation 1 for exposure to democracy, as well as exposure to democracy after independence. Table 4 indicates the least squares prediction of a year under democracy (and democracy after independence) on heights. The results show an extra year exposed to democracy while growing up increases height by 0.286 cm for Slovaks and 0.148 cm for Czechs. In other words, if the height gap between Czechs than Slovaks is 1.141 cm, an additional year under democracy reduced the height gap by 1.141–0.138 × Demage). The interaction terms were excluded for simplicity. Hence, exposure to democracy increases the heights of Slovaks more than the Czechs after controlling for ethnicity, employment, demography, income, and education.

When we split the sample by gender in columns 4 and 5, we find that only men’s height significantly changes after an additional year under a democracy. For women, we find that years exposed to a democracy while growing up did not change height among women. Finally, we excluded from the sample individuals who are over the age of 50 (as height begins to shrink around that age) and we find consistent results though coefficients are significantly smaller. An additional year growing up in a democracy increases height by 0.17 cm.

The second panel of Table 4 shows a robust and positive association between democracy post-independence and men’s heights alone, as displayed in columns 10 and 11. We find that an additional year of exposure in independent countries while growing up increase height by 0.4 cm for Slovaks and 0.2 cm for Czechs. In other words, height is 1 cm more for Czechs than Slovaks if a person was exposed to zero years under independence and this difference in height becomes smaller by 0.153 for each additional year under independence (1–0.153 x indage). Finally, restricting the sample to individuals under 50 years, although it reduced the size of the coefficient, suggests that an extra year under democracy yields a significant 0.17–0.26 cm increase in height.

## 5. Heterogeneity and Robustness

### 5.1. Income Heterogeneity

Given that heights might change in a different pattern across individual socio-economic status, we next examine whether estimates are heterogeneous by income groups. Table 5 reports the three-way interaction estimates of heights in Slovakia and the Czech Republic as well as by income tercile (only relevant coefficients are reported). Consistently with descriptive results, the effect of an extra year under democracy is larger in Slovakia—given the negative coefficient of the Czech Republic (CR) dummy variable—and is heterogeneous across income terciles. However, the negative coefficient of the two upper-income terciles suggest that height increased more than proportionally among individuals in the first tercile.

### 5.2. Robustness Checks

Our previous results cast two main doubts. First, it is unclear whether the small sample of the Czech Republic biases our results. Second, our measure of exposure to democracy might be regarded as ‘too crude’, given that ‘democratic quality’ improves over time after a transition to democracy. To address both concerns, we have carried out two additional robustness checks. More specifically, to address the potential sample selection bias, we use data from the 2005 Eurobarometer survey (Eurobarometer 64.3) which contains a similar representative sample of 1000 respondents per country, but with no sample size problems for the Czech Republic (see Appendix A). The results are summarized in Table 6 and show, consistently with previous findings, a significant and positive association between an extra year under democracy and with height. Both the coefficient for the Czech Republic (CR) and the effect size ranging from 0.18 cm to 0.31 cm among men (and no significant effect on the female sample).

As a second robustness check, we have adjusted our exposure to democracy variable by the year specific quality of democracy weight estimated by the Polity IV index for each country (Dempolity). Results are displayed in Table 7. The coefficients are very similar to those presented earlier and suggest an overall effect of a quality-adjusted year under democracy to increase heights by 0.21 to 0.28 cm. The negative coefficient of the interaction of exposure to democracy and the Czech Republic suggests a larger height change among Slovaks. The negative and significant interaction with income terciles (T_2_, T_3_) indicates that lower-income individuals (the excluded income tercile) proportionally exhibit a higher increase in heights.

## 6. Conclusions

This paper has documented a change in stature trends of both Slovaks and the Czechs after the adoption of liberal democracy and breakup from the pre-existing Czechoslovakian federation. We have drawn on two representative datasets and three measures of exposure to democracy to examine the effect of exposure to democracy on human stature. The following results emerge:

First, we estimate that every additional year exposed to democracy increases heights by 0.2–0.4 cm on average; and 0.18–0.36 cm for the sample younger than 50 years. These estimates for the sample under 50 years are more relevant, as height shrinks after the age of 50, and hence older individuals might not provide an equally accurate estimate of their stature. However, the effects are primarily driven by a change in male stature alone, consistent with previous studies [7]

Second, the transition to a liberal democracy appears to have exerted effects on heights that compare to those of other studies in East and West Germany [31] Our findings are robust to adjustments for democratic quality (weighting exposure to democracy by the year’s specific Polity IV index value). Our results suggest some evidence that the yearly change in stature after the exposure to democracy was larger in Slovakia than the Czech Republic.

Finally, we find evidence suggesting heterogeneous height changes by socio-economic status (income tercile). That is, height increases more than proportionally among individuals at the poorest income tercile. These results are consistent with the fact that social differences endure after the transition to democracy in both the Czech Republic and Slovakia, as inequalities were present already under communism [32].

It is important to point out several limitations of this study for future research to consider improving upon. First, our estimates rely on a low response rate of the WHS data for the Czech sample. The latter has led to use an alternative dataset (the Eurobarometer 64.3) which replicates WHS estimates. Hence, we believe that it is unlikely that our estimates are biased. Second, given that we rely on survey data, our results might be affected by self-reporting bias. Such a bias is unlikely to affect the relative change in heights across counties and age groups, and hence our estimates of exposure to liberal democracy. Third, one of the potential concerns of our analysis is that after the transition, there was a potential for migration of younger cohorts who might be relatively taller. However, if that were the case, it would produce a downward bias in our estimates, rendering our results as a lower bound. But more importantly, the main barriers to migration were to be lifted post-2004, and our data is predating that period. That said, there are still some outstanding concerns we have not been able to address. More specifically, we cannot separate out cohort and age effects, nor account for potential unobservable trends. Even more importantly, our results mainly report ‘robust associations’, and call for future research to use cross-country data to retrieve a causal effect of transitions to liberal democracy on stature. 

Our preferred explanation of the above findings is that the adoption of liberal democracy brought about new institutions (reduced police surveillance, socio-economic freedoms, among others) that might have reduced environmental stress, enabled human capital formation, alongside with social participation and freedom of speech, all of which would have exerted beneficial direct or indirect effects on well-being, as measured by human heights of men [33]. However, we cannot disentangle the specific mechanisms that are driving our results. The latter, as well as the specific effects of psycho-social environments, are left for future research. Our results are consistent with [19], who reported causal evidence of an effect of female enfranchisement and expansion of democracy on male human stature. On the other hand, they are likely to be underestimated as the first years of transition leads to a reduction of human stature [34]. Overall, our findings suggest that both the transition to democracy and the institutions resulting from them have real effect on wellbeing of communities.

## Figures and Tables

**Table 1:** Variable description.

Variable	Variable Description	Observations
Height (H)	Adult height in cm	2726
Democracy (D)		
Demage	Years exposed to a democracy before the age of 20	2726
DemIndyage	Years exposed to an independence before the age of 20	2726
Dempolity	Years exposed to a democracy from 1993–2003, adjusted for the “quality of democracy” with the Polity IV democracy score	2726
Controls (X)		
Gender	Dummy take the value of 1 if male; 0 if female	2726
CR	Dummy take the value of 1 Czech Republic; 0 if Slovakia	2726
Age	Categorical age group classification of individuals born between 1910–1933, 1934–1940, 1944–1953, 1954–1963, 1964–1973, 1974–1985 or younger.	2726
Employment	=1 if individual is working; 0 otherwise	2702
Income	estimated permanent income of individual	2596
Language	=1 if individual reported a language; 0 otherwise	2726

Source: [28].

**Table 2:** Mean height by gender and country.

	Slovakia		Czech Republic	
	Mean Height	Std. Dev.	Mean Height	Std. Dev.
**Women age group**			
18–29	167.69	5.68	168.55	6.53
30–39	166.32	5.66	166.17	6.38
40–49	164.63	5.98	166.39	7.28
50–59	164.09	5.74	164.35	5.35
60–69	161.83	5.80	162.70	5.53
70–98	160.70	5.30	162.58	5.81
**Men age group**				
18–29	180.79	7.44	180.24	7.46
30–39	178.88	7.27	180.84	6.95
40–49	178.61	6.85	178.52	7.45
50–59	175.82	5.67	176.09	6.51
60–69	171.67	6.67	174.92	6.16
70–98	172.00	6.91	171.83	6.14

Note: The table above reports the average height (and standard deviation) by gender and age group in the Czech Republic and Slovakia. Source: [28] Std. Dev refers to standard deviation.

**Table 3:** Average height by income terciles (T_i_) *i* = 1,2,3, gender (Male, Female) and country (sk = Slovakia, cz = Czech Republic).

Variable.	Obs	Mean	Std. Dev.	Min	Max
First Income Tercile (T_1_)					
T_1__female_sk	408	164.62	2.24	160.51	167.42
T_1__male_sk	143	176.78	3.97	166.12	180.24
T_1__female_cz	190	164.41	2.49	161.34	168.36
T_1__male_cz	133	175.26	4.06	169.83	181.9
Second Income Tercile (T_2_)					
T_2__female_sk	352	166.12	2.04	163.06	168.23
T_2__male_sk	202	178.59	1.37	173	179.93
T_2__female_cz	185	164.82	1.47	162.75	166.77
T_2__male_cz	140	177.01	2.22	173.95	179.81
Third Income Tercile (T_3_)					
T_3__female_sk	380	166.07	1.22	159.67	167.66
T_3__male_sk	234	180.31	1.73	174.67	181.36
T_3__female_cz	123	167.08	2.44	162.8	170.75
T_3__male_cz	130	180.20	2.76	173.78	183.43

Note: This table reports the average male and female heights of individuals in the Czech Republic and Slovakia by income tercile (T_1_, T_2_, T_3_). Source: [28].

**Table 4:** Ordinary least squares (OLS) regressions of exposure to democracy (Demage = years lived under democracy, DemIndyage = exposure to democracy after independence) on individuals height.

Dmocracy	(1)	(2)	(3)	(4)	(5)	(6)
	All	All	All	Male	Female	Under 50
Demage	0.264 ***	0.217 ***	0.286 ***	0.273 **	0.107	0.174 **
	(0.0714)	(0.0735)	(0.0783)	(0.134)	(0.0883)	(0.0757)
Constant	165.0 ***	165.0 ***	164.7 ***	168.1 ***	166.1 ***	166.9 ***
	(0.801)	(0.825)	(0.834)	(7.592)	(5.185)	(0.853)
Observations	2726	2596	2596	967	1605	1816
R-squared	0.569	0.576	0.577	0.261	0.141	0.563
Democracy After Independence						
	(7)	(8)	(9)	(10)	(11)	(12)
	All	All	All	Male	Female	Under 50
DemIndyage	0.318 ***	0.269 ***	0.345 ***	0.382 ***	0.0804	0.257 ***
	(0.0790)	(0.0809)	(0.0882)	(0.147)	(0.0986)	(0.0807)
Constant	165.4 ***	165.3 ***	165.0 ***	166.8 ***	167.9 ***	167.0 ***
	(0.659)	(0.675)	(0.685)	(5.136)	(7.527)	(0.704)
Observations	2726	2596	2596	967	1605	1805
R-squared	0.570	0.577	0.578	0.264	0.141	0.564
Ethnicity	No	No	Yes	Yes	Yes	Yes
Employment	No	No	Yes	Yes	Yes	Yes
Demographic	Yes	Yes	Yes	Yes	Yes	Yes
Education	Yes	Yes	Yes	Yes	Yes	Yes
Income terciles	No	No	No	Yes	Yes	Yes

Note: This table reports regression coefficients of an extra year of exposure to democracy (Demage) and exposure to democracy after independence (DemIndyage), respectively, on heights (measured in cm) and controlling for gender, income, ethnicity, employment, and education. CR = Czech Republic dummy being the excluded category SK = Slovakia. Standard errors in parentheses; *** *p* < 0.01, ** *p* < 0.05, * *p* < 0.1.

**Table 5:** Regressions measuring the exposure to democracy by income tercile (T) and Country (CR, SK).

Democracy x Tercile	(1)	(3)	(3)
	Demoage	Demoage only under 50	DemIndyage
D	0.432 ***	0.356 ***	0.432 ***
	(0.110)	(0.123)	(0.110)
CR × D	−0.251 **	−0.295**	−0.251 **
	(0.105)	(0.125)	(0.105)
D × T_2_	−0.431 **	(1.218)	−0.431 **
	(0.186)	−0.188	(0.186)
D × T_3_	−0.465 **	(0.127)	−0.465 **
	(0.184)	−0.233 *	(0.184)
CR × D × T_2_	0.0340	(0)	0.0340
	(0.140)	0.0854	(0.140)
CR × Demage × T_3_	0.379 ***	(0.163)	0.379 ***
	(0.137)	0.397 **	(0.137)
Constant	161.8 ***	162.2 ***	161.8 ***
	(4.439)	(4.504)	(4.439)
Ethnicity	Yes	Yes	Yes
Employment	Yes	Yes	Yes
Demographic	Yes	Yes	Yes
Education	Yes	Yes	Yes
Income qiantile controls	Yes	Yes	Yes
Observations	2596	1805	2572
R-squared	0.579	0.564	0.583

Note: This table reports OLS regression coefficients measuring the change in heights of an extra year of exposure to democracy (Demage, DemIndyage) by income tercile (T_2_, T_3_, and T_1_ being the excluded category) and country (Czech Republic = CR, and SK = Slovakia being the excluded category) on heights (measured in cm). Standard errors in parentheses *** *p* < 0.01, ** *p* < 0.05, * *p* < 0.1.

**Table 6:** Regression estimates using Eurobarometer survey (Eurobarometer 64.3).

Variables	(1)	(2)	(3)	(4)
	All	Men	All	Men
Demage	0.184 **	0.313 ***		
	(0.0752)	(0.0922)		
DemIndyage			0.224 ***	0.357 ***
			(0.0840)	(0.101)
CR	1.341 ***	1.049 ***	1.344 ***	1.066 ***
	(0.277)	(0.341)	(0.277)	(0.341)
Constant	164.7 ***	163.3 ***	164.9 ***	163.8 ***
	(0.992)	(1.197)	(0.858)	(1.017)
Controls	Yes	Yes	Yes	Yes
Observations	1986	1144	1986	1144
R-squared	0.564	0.134	0.564	0.134

Note: This table reports regression coefficients of an extra year of exposure to democracy (Demage) and exposure to democracy after independence (DemIndyage), respectively, on heights (measured in cm) using the Eurobarometer 64.3 survey data, and controlling for gender, income, ethnicity, employment, and education. CR = Czech Republic dummy being the excluded category SK = Slovakia. Standard errors in parentheses *** *p* < 0.01, ** *p* < 0.05, * *p* < 0.1.

**Table 7:** Regression measuring the exposure to democracy adjusted by “quality.”.

Quality Adjusted Democracy	(1)	(2)	(3)	(4)
	All	All	All	All
Dempolity	0.280 ***	0.230 ***	0.217 **	0.660 ***
	(0.0833)	(0.0854)	(0.0881)	(0.173)
CR × Dempolity				−0.448 ***
				(0.168)
				(0.885)
CR × Dempolity × T_2_				−0.431 **
				(0.186)
CR × Dempolity × T_3_				−0.465 **
				(0.184)
Constant	166.0 ***	165.9 ***	165.9 ***	162.1 ***
	(0.599)	(0.615)	(0.616)	(4.394)
Ethnicity	No	No	Yes	Yes
Employment	No	No	Yes	Yes
Demographic	Yes	Yes	Yes	Yes
Education	Yes	Yes	Yes	Yes
Income terciles	No	No	Yes	Yes
Observations	2726	2596	2596	2572
R-squared	0.569	0.576	0.576	0.584

Note: This table reports OLS regression coefficients of an extra year of exposure to democracy but weighting each years of exposure by the polity index of the year (Dempolity) on heights (measured in cm), as well as the interaction with each country (CR = Czech Republic and SK = Slovakia being the omitted category) and income tercile (T_2_, T_3_, and T_1_ being the excluded category). Standard errors in parentheses. *** *p* < 0.01, ** *p* < 0.05, * *p* < 0.1.

**Figure A1:**
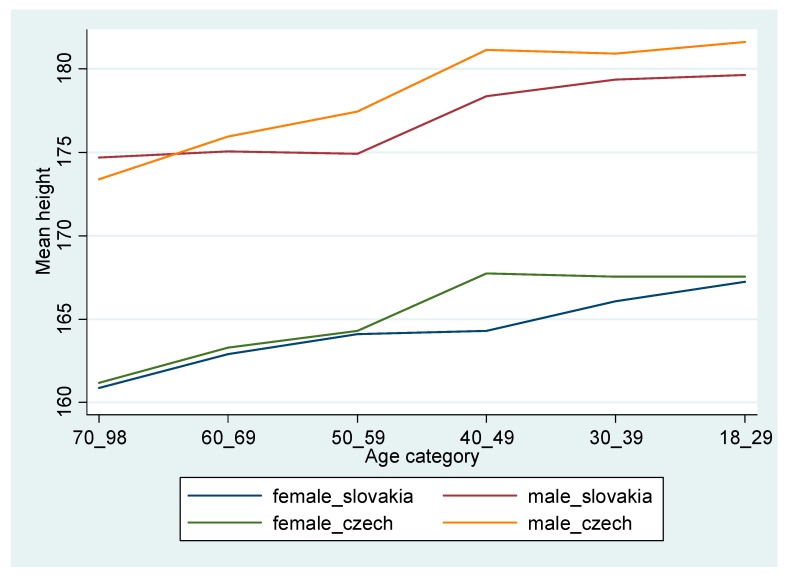
Height by age group, gender, and country, 2005 Eurobarometer 2005 data [27].

## Appendix A

**Table A1:** Descriptive Statistics – World Health Survey 2003.

		*N*	Mean	SD
Height	Height in cm	1917	171.632	9.280
CR	Czech Republic	2412	0.207	
Demage	Years under democracy	2412	4.962	5.335
DemIndyage	Years under independece	2412	3.355	4.12
Income	Composite income	1827	0.411	0.979
Language	Language dummy	2412	0.795	
Gender	Men	2412	0.408	
Age	Age in years	2412	32.37	9.46
No education		1926	0.633	
Primary Education		1926	0.242	
Secondary Education		1926	0.541	
Upper Education		1926	0.152	
Employed		1914	0.680	

Note: This tables provides descriptive statistics of the variables employed from the World Health Survey 2003. N refers to the number of observations, Mean refers to the mean of the variable and Std. Dev refers to the standard deviation for continuous variables [28].

**Table A2:** Descriptive statistics Eurobarometer 2005 data.

Variable	N	Mean	Std. Dev.
Height	1986	170.721	9.1274
Age	2024	46.111	16.181
CR	2024	0.492	
Gender	2024	0.420	
Demage	2024	2.670	4.760
DemIndyage	2024	1.777	3.656
Age 18–29	2024	0.191	
Age 30–39	2024	0.190	
Age 40–49	2024	0.186	
Age 50–59	2024	0.195	
Age 60–69	2024	0.159	
Age 70–98	2024	0.077	

Note: This tables provides descriptive statistics of the variables employed from the Eurobarometer 2005 data. N refers to the number of observations, Mean refers to the mean of the variable and Std. Dev refers to the standard deviation for continuous variables [27].
